# Impacts of a social and behavior change communication program implemented at scale on infant and young feeding practices in Nigeria: Results of a cluster-randomized evaluation

**DOI:** 10.1371/journal.pone.0277137

**Published:** 2022-12-08

**Authors:** Valerie L. Flax, Mariam Fagbemi, Courtney H. Schnefke, Auwalu A. Kawu, Susan Edwards, Jennifer Unangst, Sujata Bose

**Affiliations:** 1 RTI International, Research Triangle Park, North Carolina, United States of America; 2 Kantar, Lagos, Nigeria; 3 Alive & Thrive Nigeria, FHI Solutions, Abuja, Nigeria; 4 Alive & Thrive, FHI Solutions, Washington, District of Columbia, United States of America; State University of Rio de janeiro, BRAZIL

## Abstract

**Background:**

Infant and young child feeding (IYCF) practices are important for child survival and healthy growth, but IYCF practices remain suboptimal in Nigeria. The objective of this study was to measure the impact of Alive & Thrive’s IYCF social and behavior change communication intervention on early initiation of breastfeeding, exclusive breastfeeding, and minimum dietary diversity in Kaduna and Lagos States.

**Methods:**

Local government areas were randomly allocated to intervention or comparison. Cross-sectional surveys of households with children aged 0–23 months were conducted [N = 6,266 baseline (2017), N = 7,320 endline (2020)]. Logistic regression was used to calculate difference-in-differences estimates (DDEs) of impact on IYCF practices and to assess within group changes from baseline to endline. Associations between intervention exposures and IYCF practices were tested in both study groups combined.

**Results:**

In Kaduna, a positive differential effect of the intervention was found for exclusive breastfeeding (adjusted DDE 8.9 pp, *P*<0.099). Increases in both study groups from baseline to endline were observed in Kaduna for early initiation of breastfeeding (intervention 12.2 pp, *P* = 0.010; comparison 6.4 pp, *P* = 0.118) and minimum dietary diversity (intervention 20.0 pp, *P*<0.001; comparison 19.7 pp, *P*<0.001), which eliminated differential effects. In Lagos, no differential intervention impacts were found on IYCF practices because changes in early initiation of breastfeeding from baseline to endline were small in both study groups and increases in both study groups from baseline to endline were observed for exclusive breastfeeding (intervention 8.9 pp, *P* = 0.05; comparison 6.6 pp, *P*<0.001) and minimum dietary diversity (intervention 18.9 pp, *P*<0.001; comparison 24.3 pp, *P*<0.001). Odds of all three IYCF practices increased with exposure to facility-based interpersonal communication in both states and with community mobilization or mass media exposure in Kaduna.

**Conclusions:**

This evaluation found weak impacts of the Alive & Thrive intervention on IYCF practices in the difference-in-differences analysis because of suspected intervention spillover to the comparison group. Substantial within group increases in IYCF practices from baseline to endline are likely attributable to the intervention, which was the major IYCF promotion activity in both states. This is supported by the association between intervention exposures and IYCF practices.

**Trial registration:**

The study was registered with clinicaltrials.gov (NCT02975063).

## Introduction

Optimal infant and young child feeding (IYCF) practices are essential for child survival, growth, and development [1]. The World Health Organization (WHO) recommends that children start breastfeeding within 1 hour of delivery (early initiation), are exclusively breastfed until 6 months of age, and receive a minimally diverse diet that includes at least 4 of 7 food groups daily [2]. According to current UNICEF estimates for West Africa, 47% of infants start breastfeeding within 1 hour, 34% of infants aged 0 to 5 months are exclusively breastfed, and 23% of children aged 6 to 23 months achieve minimum dietary diversity [3]. Nigeria is the most populous country in Africa, with approximately 200 million inhabitants [4]. It has great variability in health and nutrition indicators across different regions of the country [5] and some of Africa’s most densely populated urban areas. Nationally in 2018, the prevalence of key IYCF indicators in Nigeria was 42% for early initiation of breastfeeding, 29% for exclusive breastfeeding, and 25% for minimum dietary diversity [5]. Although these percentages represent increases from 2013 [6], Nigeria remains below the average for West Africa on breastfeeding indicators and on par with the region for dietary diversity, indicating an important gap in IYCF practices.

Small-scale interventions in low- and middle-income countries (LMICs) have shown that interpersonal communication and social support for mothers from health workers or family members are effective for improving breastfeeding practices [7–9]. Several studies that provided mothers in LMICs with nutrition education or interpersonal communication related to complementary feeding also showed that such interventions can increase maternal knowledge and improve practices [10–18]. More recently, studies in Bangladesh, Vietnam, Ethiopia, and Burkina Faso tested IYCF interventions implemented by Alive & Thrive—a global nutrition initiative to save lives, prevent illness, and ensure healthy growth of mothers and children—at scale using a model that includes interpersonal communication, community mobilization, and mass media. These interventions, adapted to the context in each country, were effective at increasing optimal IYCF feeding practices [15, 16, 19–23]. Alive & Thrive used the evidence generated for further scale up within each country and region and to fill gaps in global knowledge related to IYCF programming. In Nigeria, Alive & Thrive in coordination with the Federal Ministry of Health purposefully selected two states—Kaduna and Lagos—that had different religious and ethnic profiles and levels of urbanization. The evaluation in Nigeria included state-level analysis, which differs from previous Alive & Thrive evaluations and allows for evidence generation on how the intervention worked in varied settings.

The main aim of this study was to assess the impact of the Alive & Thrive IYCF intervention model on early initiation of breastfeeding, exclusive breastfeeding, and minimum dietary diversity in Kaduna and Lagos States. With IYCF mass media supported by Alive & Thrive available throughout the two states, we hypothesized that the interpersonal communication and community mobilization intervention components would increase the three target IYCF practices in the intervention group versus the comparison group. The secondary aims of the study were to assess the impact of the intervention on maternal knowledge and awareness of IYCF practices and the association of maternal intervention exposure with recommended IYCF practices.

## Methods

### Study setting and design

Alive & Thrive’s intervention was implemented in Kaduna and Lagos States, Nigeria. Kaduna State is in the northwestern part of Nigeria and has a population of approximately 9 million, with about 20% living in urban areas [24]. Kaduna has a mix of Muslim and Christian religions and more than 60 ethnic groups, the most prominent being Hausa and Fulani. Lagos State is a large urban area on the southwest coast of Nigeria with a population of more than 15 million; although by some estimates it has up to 25 million inhabitants [25]. Lagos is the economic capital of Nigeria. It is a melting pot of ethnic groups, with Yoruba and Pidgin as the main languages.

This study was a cluster-randomized impact evaluation of Alive & Thrive’s intervention to improve IYCF practices in Kaduna and Lagos States. All 23 local government areas (LGAs), the largest administrative subunits within a state, in Kaduna and all urban LGAs (16 out of 20) in Lagos were included in the study. A statistician at RTI International, the external evaluation partner, initially randomly allocated LGAs to three study groups (two different intervention groups and a comparison group) with a computer program using approximately a 1:1:1 allocation ratio. The LGAs were stratified by zonal region (Central, North, and South) and urbanicity (rural/small semiurban, urban/large semiurban) in Kaduna and region (Central, East, and West) and population size (small, large) in Lagos. After randomization, Alive & Thrive in collaboration with the Ministries of Health in the two states opted for a single intervention group and decided to maintain the original plan for two-thirds of the LGAs in each state to receive the intervention. This led the evaluators to merge the two randomized intervention groups into one group, resulting in two-thirds of the LGAs assigned to the intervention group and one-third to the comparison group (2:1 ratio). To measure intervention impacts, cross-sectional population-based surveys were conducted before and after the intervention in 2017 and 2020, respectively. Neither survey participants nor data collectors were blinded to study arm assignment.

### Intervention

The theory of change for the intervention in Nigeria (S1 File) is built on the socio-ecological model and was tested by Alive & Thrive in other countries [26]. The theory posits that interpersonal communication and mass media contacts with mothers and family members and community mobilization through religious and community leaders, in conjunction with advocacy and strategic use of data, will lead to improved knowledge and optimal IYCF practices, better health worker capacity to deliver quality IYCF services, and improved health outcomes [27].

Interpersonal communication and community mobilization activities were conducted in 16 intervention LGAs in Kaduna State and 10 intervention LGAs in Lagos State. In collaboration with state Ministries of Health, Alive & Thrive trained 1,554 traditional birth attendants (TBAs), 4,892 health workers at public and private health facilities, and 123 master trainers who could support cascade training to other health facilities in the intervention LGAs. The training lasted for 2 days and covered IYCF practices, interpersonal communication skills and counseling techniques, one-on-one communication, facilitation skills for group counseling, and data reporting. Health providers were supported with posters (15,000 Kaduna, 10,000 Lagos), leaflets (20,000 each state), and IYCF messages presented on LED screens where available in health facilities. Health providers delivered IYCF messages to pregnant women and mothers with a child 0–23 months of age through group and individual interpersonal communication at health facilities during antenatal, postnatal, and well- and sick-child visits. In addition, 479 community volunteers (Kaduna 228, Lagos 251), including representatives from community-based organizations (such as market associations, religious groups, women’s groups, and professional associations) and community leaders, were trained to conduct home visits and hold community meetings to discuss IYCF with pregnant women, mothers with young children, and men. No specific number or timing of facility- or community-based contacts for women or other community members was predetermined.

Alive & Thrive developed and produced key IYCF mass media messages in Hausa and English languages through the “Start Strong” campaign to promote early initiation of breastfeeding, exclusive breastfeeding, and dietary diversity. These were deployed through television and radio (https://www.aliveandthrive.org/en/resources/start-strong-for-a-better-future-tv-spots) and complemented with posters, leaflets, billboards, and vehicle ads (https://www.aliveandthrive.org/en/resources/helping-infants-in-nigeria-start-strong-for-a-better-future; https://www.aliveandthrive.org/en/resources/start-strong-for-a-better-future-posters). The mass media campaign was implemented for 10 months from September 2019 through June 2020. Television ads were shown on two stations in Kaduna and three in Lagos and on three cable channels available nationwide, with more than 1,500 spots in Kaduna and more than 2,000 in Lagos. Radio ads were aired on three stations in Kaduna and four in Lagos, with more than 6,000 spots in Kaduna and close to 8,000 in Lagos. Television and radio ads were aired 7–8 times per day. Television and radio call-in programs amplified the messages.

Women in the comparison LGAs were expected to receive usual standard-of-care counseling on IYCF during health facility visits. Alive & Thrive did not support training on IYCF for health providers or community mobilization activities related to IYCF in the comparison LGAs. Start Strong television and radio ads were aired throughout the two states and women in both study groups could have been exposed to them. Therefore, the evaluation was designed to measure the impact of interpersonal communication and community mobilization in a setting where mass media on IYCF was widely available.

### Sampling

Two sampling methods were used for the surveys. The primary method was geo-sampling, in which a geographic information system (GIS) grid was overlaid on the map of each LGA. Primary sampling units (1 km^2^) were randomly selected then secondary sampling units (50 m^2^ cells in urban areas, 100 m^2^ cells in semiurban areas, and 150 m^2^ in rural areas) were randomly selected within the primary sampling units. All households within the secondary sampling units were enumerated using a household screener with the head of household or senior female household member and all eligible women within the secondary sampling units were interviewed. This method was supplemented with random route walks in LGAs where an adequate sample in some subgroups was not attained through geo-sampling alone. The random route walks were conducted in 9 LGAs at baseline and 8 LGAs at endline in administrative wards that did not overlap with primary or secondary sampling units from the geo-sample.

### Sample size

At baseline, sample size calculations were performed to determine the target number of completed interviews, estimated at 268 mothers per state subgroup (Kaduna rural, Kaduna urban, and Lagos), intervention group (intervention/comparison), and for each child age group (mothers with a child aged 0 to 5 months; mothers with a child aged 6 to 23 months). This estimate assumed the evaluation would be conducted across the two states using a one-sided test for differences between two independent sample proportions with a significance level of 0.05, power of 80%, a design effect of 2.0 to account for the clustered sample design, and a desired minimum detectable difference of 13 percentage points for the three main outcome variables, based on trends in national estimates of IYCF indicators over time [6, 28] and differences detected in Alive & Thrive evaluations in other countries [15, 19, 20]. A design effect of 2.0 was chosen because it was considered sufficiently conservative to account for the unknown clustering effects of using a geo-sampling approach and was expected to account for the combined impacts of variation in cluster sizes and within-cluster homogeneity. The total target number of completed interviews for baseline was 3,216 across the two states and all intervention subgroups and analysis domains. After the baseline survey was completed, the sample sizes were reconsidered for the endline to support the state-level analysis. The sample size calculations were redone using the same approach previously described but accounting for the observed baseline sample sizes, sample size imbalance between the intervention and comparison groups, and the desire to power state-level estimates. The revised target number of completed interviews for endline was 4,348 (1,976 in Kaduna; 2,372 in Lagos). Target sample size tables for baseline and endline are shown in S2 File.

### Data collection

The data were collected by separate teams of trained enumerators and supervisors in each state. The questionnaire was available in Hausa, Yoruba, and English. Trained interviewers recruited mothers and administered the questionnaire to them at their homes using the NField tablet-based data collection system. Mothers were eligible to participate if they had a biological child aged 0 to 23 months and were 18 years and older or 15 to 17 years old and married. Younger mothers are considered emancipated under Nigerian law and allowed to consent for themselves if they are married.

The baseline survey was conducted from January to July 2017 and the endline survey was conducted from January to December 2020. The duration of endline data collection was elongated because of a pause from March through August 2020 necessitated by the COVID-19 pandemic.

Ethical approval for the evaluation was obtained from institutional review boards at RTI and FHI 360, and from the ethics committees of the Kaduna State Ministry of Health and the Lagos State Government through the Lagos State University Teaching Hospital, Ikeja. The original protocol approved by RTI is found in S3 File. Verbal informed consent was obtained from all study participants. Mothers were given an incentive of 500 Naira of cell phone airtime. The study was registered with clinicaltrials.gov (NCT02975063).

### Measures

The household survey questionnaire was based on the UNICEF conceptual framework for improved nutrition [29] and guided by the questionnaires used in other Alive & Thrive evaluations [19]. The questionnaire captured data on IYCF practices, mothers’ IYCF knowledge and awareness, exposure to the intervention, and participant and household characteristics. The three main outcomes were measured using the WHO IYCF questionnaire and calculated as binary variables as per WHO guidelines [30]. They were defined as follows: early initiation of breastfeeding (starting to breastfeed within 1 hour of delivery), exclusive breastfeeding (giving an infant aged 0 to 5 months only breast milk on the previous day), and minimum dietary diversity (giving a child aged 6 to 23 months 4 or more food groups on the previous day) [30]. Knowledge of optimal IYCF practices was determined through questions where the enumerator did not read out response options, whereas awareness was based on questions where the participant was asked if she had ever heard of particular IYCF practices. Knowledge questions had multiple responses and were categorized as binary variables (correct/incorrect) for analysis. Awareness variables were also analyzed in a binary format (ever heard/never heard). Exposure to Start Strong mass media—including IYCF television and radio ads, posters, and billboards—was collected only at endline because the mass media campaign was developed and implemented during the program. Socioeconomic characteristics were measured using standard questions from the Nigeria Demographic and Health Survey (DHS) [6] and the Household Food Insecurity Access Scale (HFIAS) [31]. HFIAS was used to calculate household food insecurity access categories. A list of 39 household assets (yes/no) were summed to create an asset score.

### Data analysis

Analyses were conducted using Stata MP version 16 with survey commands to account for clustering at the level of the LGA. Descriptive statistics were calculated by study group and state for participants’ socioeconomic characteristics. Difference-in-differences estimates (DDEs) of the program’s impact on IYCF practices, knowledge, and awareness in each state were calculated using logistic regression models including treatment (intervention/comparison), time (baseline/endline), and a treatment by time interaction term [32]. Adjusted difference-in-differences models controlled for child’s age and sex. Based on conversations with Alive & Thrive staff about possible intervention spillover, a within-group analysis was performed using logistic regression models to test for changes from baseline to endline within study groups in each state.

An exposure analysis was conducted using separate models for each main outcome and state to determine which exposure variables were associated with the outcomes. Having a caesarean section was controlled for in the early initiation of breastfeeding models. Three types of exposures were tested–interpersonal communication at a health facility, community mobilization, and mass media.

To better understand the effects of the COVID-19 pandemic on the endline results, a sensitivity analysis was conducted to assess the impact of the pandemic on our main outcome measures. Inverse probability weights (IPW) were used to adjust for differences between the endline sample collected before and after COVID restrictions in each state. Propensity models for being in the after COVID sample were fit for each state and adjusted for clustering at the level of the LGA. Each model controlled for variables that differed before compared with during the COVID pandemic, including child’s age category (0–5, 6–11, 12–17, 28–23 months), number of adults in household, type of fuel used for cooking, and household food insecurity access category (secure, mild, moderate, severe). The type of fuel variable had several response options. Only responses with more than 10% of the sample response before COVID were included in the model. The Lagos model accounted for natural gas and kerosene cooking fuels. The Kaduna model accounted for charcoal and wood cooking fuels.

## Results

**Fig 1:**
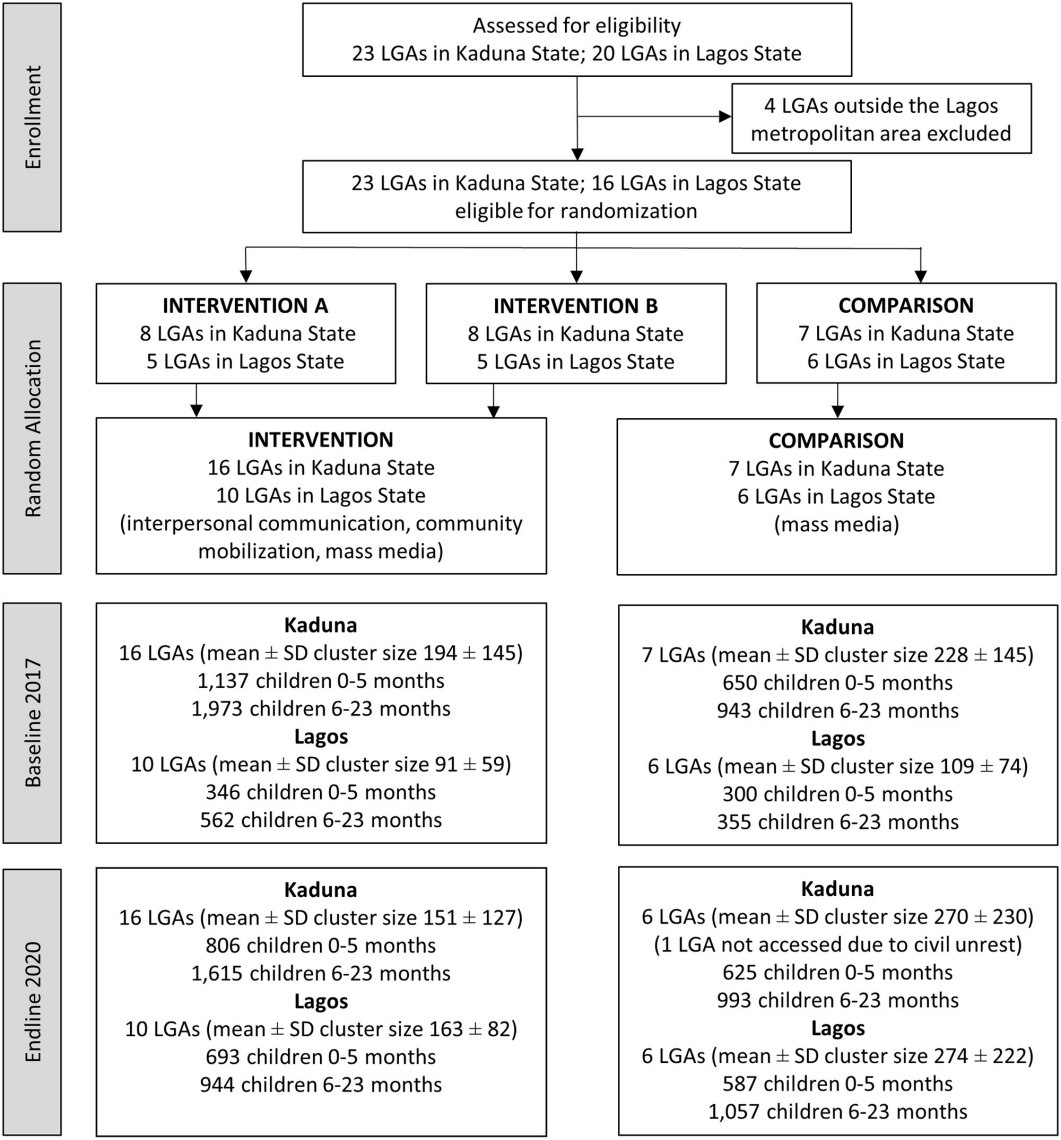
Consort diagram for the Alive & Thrive Nigeria impact evaluation.

The CONSORT flowchart for the study (Fig 1) shows the LGA allocation, cluster sizes, and final household sample sizes for subgroups by child’s age. The intervention was implemented in all intervention LGAs as planned; however, survey data collection at both baseline and endline was not possible in parts of some LGAs in Kaduna State because of civil unrest, resulting in population movement and insecurity. Two intervention LGAs and two comparison LGAs in Kaduna were not fully completed at baseline. Four intervention LGAs were not fully completed and one comparison LGA was not visited at all at endline. Two of the intervention LGAs and the comparison LGA not completed at endline were the same as at baseline. Among eligible households at baseline, 1.7% refused to participate and 1.7% did not complete their interviews in Kaduna; 1.7% refused to participate and 0.4% did not complete their interviews in Lagos. Among eligible households at endline, 2.0% refused to participate and 0.5% did not complete their interviews in Kaduna; 3.6% refused to participate and 3.1% did not complete their interviews in Lagos.

### Household and participant characteristics

Household and participant characteristics are shown in Table 1. Characteristics were well balanced across intervention and comparison groups at each time point. In Kaduna, more mothers in both study groups had never attended school and fewer mothers in both study groups reported that they were housewives at baseline than endline. In Lagos, characteristics were similar at baseline and endline.

**Table 1:** Household and participant characteristics, by state^1^.

	**BASELINE**				**ENDLINE**			
	**Intervention**		**Comparison**		**Intervention**		**Comparison**	
	**Mean**	**SE**	**Mean**	**SE**	**Mean**	**SE**	**Mean**	**SE**
**KADUNA**	(N = 3110)		(N = 1593)		(N = 2421)		(N = 1618)	
Household size	7.1	0.4	6.5	0.5	5.6	0.2	5.9	0.2
Number of adults (≥18 years)	2.8	0.1	2.6	0.2	2.3	0.0	2.3	0.1
Number of children <5 years	1.9	0.1	1.9	0.1	1.6	0.0	1.7	0.0
Household assets (29 items)	7.1	0.5	7.5	0.8	6.3	0.5	6.3	0.6
Mother’s age	26.8	0.4	26.6	0.4	26.5	0.3	26.0	0.6
Child’s age	9.5	0.4	8.9	0.3	9.7	0.2	9.1	0.3
	**%**	**N**	**%**	**N**	**%**	**N**	**%**	**N**
Household food insecurity access category								
Food secure	52.9	1608	53.8	845	56.2	1361	55.1	892
Mild food insecure access	6.6	202	7.4	117	8.6	208	7.8	126
Moderate food insecure access	14.5	442	13.3	209	15.9	386	16.1	261
Severe food insecure access	25.9	787	25.5	401	19.2	466	21.0	339
Mother’s education								
Never attended school	40.0	1243	39.4	628	21.5	439	24.7	283
Primary school	22.0	683	21.3	340	29.0	592	31.3	358
Secondary school	32.3	1005	31.8	506	41.5	848	37.4	428
Vocational training	0.1	2	0.1	1	0.3	7	0.3	3
College or higher	5.6	174	7.4	118	7.6	155	6.4	73
Mother’s marital status (married)	97.6	3035	97.9	1559	98.1	2376	99.1	1603
Mothers’ occupation								
Farmer	5.4	169	2.5	40	3.2	78	2.5	40
Salaried, government	1.3	39	1.5	24	1.3	32	1.2	19
Salaried, nongovernment	2.0	62	2.0	32	1.3	31	1.4	22
Small trader/self-employed	57.0	1772	54.4	867	54.9	1325	54.8	884
Housewife	27.9	867	20.9	492	36.4	879	38.8	626
Retired	0.1	3	0.1	2	0.0	1	0.0	0
Unemployed	5.3	165	6.8	108	1.5	36	0.6	10
Student	1.0	32	1.8	29	1.2	30	0.8	13
Sex of the index child (male)	51.0	1584	51.4	819	49.2	1191	48.6	786
	**Mean**	**SE**	**Mean**	**SE**	**Mean**	**SE**	**Mean**	**SE**
**LAGOS**	(N = 908)		(N = 655)		(N = 1637)		(N = 1644)	
Household size	4.5	0.1	4.3	0.1	4.2	0.0	4.2	0.0
Number of adults (≥18 years)	2.2	0.0	2.2	0.0	2.1	0.0	2.1	0.0
Number of children <5 years	1.4	0.0	1.4	0.0	1.4	0.0	1.4	0.0
Household assets (29 items)	10.4	0.2	10.3	0.6	10.9	0.3	11.8	0.2
Mother’s age	29.8	0.3	30.2	0.4	30.0	0.3	30.9	0.0
Child’s age	9.2	0.4	8.0	1.1	8.9	0.5	9.6	0.4
	**%**	**N**	**%**	**N**	**%**	**N**	**%**	**N**
Household food insecurity access category								
Food secure	51.6	456	60.7	385	56.0	916	58.3	958
Mild food insecure access	6.5	57	6.3	40	9.0	147	10.1	166
Moderate food insecure access	21.6	191	16.9	107	17.3	284	16.4	270
Severe food insecure access	20.3	179	16.1	102	17.7	290	15.2	250
Mother’s education								
Never attended school	2.4	22	1.5	10	1.4	23	0.6	10
Primary school	11.6	105	6.6	43	7.1	115	5.6	92
Secondary school	64.2	583	63.2	414	65.0	1059	57.4	941
Vocational training	0.1	1	0.3	2	0.9	14	1.8	29
College or higher	21.7	197	28.4	186	25.7	418	34.6	566
Mother’s marital status (married)	96.9	879	98.5	645	98.0	1603	98.3	1616
Mother’s occupation								
Salaried, government	1.3	12	2.1	14	1.7	28	1.9	31
Salaried, nongovernment	8.0	73	9.9	65	8.7	141	11.5	188
Small trader/self-employed	67.4	611	70.5	462	69.1	1122	67.0	1094
Housewife	16.1	146	13.9	91	18.7	303	17.3	282
Unemployed	6.0	54	2.6	17	1.8	29	1.7	27
Student	1.2	11	0.9	6	0.1	1	0.7	11
Sex of the index child (male)	48.7	442	49.9	327	50.7	830	53.1	873

^1^Estimates account for clustering at the level of the LGA.

### IYCF practices

In Kaduna, a positive differential impact of the intervention was detected for exclusive breastfeeding (unadjusted DDE 11.3, *P* = 0.112; adjusted DDE 8.9 pp, *P* = 0.099), but no effects were detected for early initiation of breastfeeding or minimum dietary diversity (Table 2). Increases in both study groups from baseline to endline were observed for early initiation of breastfeeding (intervention 12.2 pp, *P* = 0.010; comparison 6.4 pp, *P* = 0.118) and minimum dietary diversity (intervention 20.0 pp, *P*<0.001; comparison 19.7 pp, *P*<0.001), which limited the possibility of detecting differential intervention effects.

**Table 2:** Impact of Alive & Thrive on IYCF practices in Kaduna and Lagos States, Nigeria.

	Baseline				Endline				Intervention T_2_-T_1_ pp difference	Comparison T_2_-T_1_ pp difference	Unadjusted DDE^1^ pp (95% CI)	Adjusted DDE^2^ pp (95% CI)
	Intervention		Comparison		Intervention		Comparison					
	%	N/Total N	%	N/Total N	%	N/Total N	%	N/Total N				
**KADUNA**												
Early initiation of breastfeeding	34.6	1064/3071	35.0	552/1576	46.9	1124/2398	41.4	663/1602	12.2*	6.4	5.9 (-5.7, 17.5)	5.9 (-5.6, 17.5)
Exclusive breastfeeding	27.1	308/1137	28.0	182/650	43.5	351/806	33.1	207/625	16.5*	5.1***	11.3 (-2.8, 25.4)	8.9 (-1.8, 19.6)†
Minimum dietary diversity	20.5	405/1973	19.1	180/943	40.5	654/1615	38.8	385/993	20.0***	19.7***	0.3 (-8.3, 8.9)	0.4 (-7.2, 25.5)
**LAGOS**												
Early initiation of breastfeeding	40.2	360/896	44.3	289/653	43.5	698/1605	38.5	623/1619	3.3	-5.8	9.1 (-9.5, 27.7)	8.2 (-9.2, 25.5)
Exclusive breastfeeding	54.6	189/346	59.3	178/300	63.5	440/693	65.9	387/587	8.9*	6.6***	2.3 (-6.6, 11.2)	1.3 (-5.0, 7.6)
Minimum dietary diversity	39.7	223/562	35.2	125/355	58.6	553/944	59.5	629/1057	18.9***	24.3***	-5.4 (-16.9, 6.1)	-2.9 (-15.1, 9.4)

DDE, difference-in-differences estimate; pp, percentage point; T_1_, time 1 (baseline); T_2_, time 2 (endline)

^1^Unadjusted DDE accounts for clustering at the level of the LGA.

^2^Adjusted DDE accounts for clustering at the level of the LGA and adjusts for child’s age and sex.

^†^*P*<0.1,

*P<0.05,

**P<0.01,

***P<0.001

In Lagos, no differential impacts of the intervention were found on the three main outcomes. The lack of effects can be attributed to small changes from baseline to endline in both study groups for early initiation of breastfeeding and to similar size changes from baseline to endline in both study groups for exclusive breastfeeding (intervention 8.9 pp, *P* = 0.049; comparison 6.6 pp, *P*<0.001) and minimum dietary diversity (intervention 18.9 pp, *P*<0.001; comparison 24.3 pp, *P*<0.001).

### Mothers’ IYCF knowledge and awareness

Based on questions where response options were not read to mothers, in Kaduna, a negative differential impact of the intervention was found for mothers’ knowledge of dietary diversity (unadjusted DDE -21.3 pp, *P* = 0.045; adjusted DDE -21.3 pp, *P* = 0.046) (Table 3). This effect resulted from a decrease in knowledge in the intervention group and an increase in the comparison group from baseline to endline (intervention -16.0 pp, *P* = 0.024; comparison 5.4 pp, *P* = 0.473). No effects were detected on other knowledge variables because mothers’ knowledge of early initiation of breastfeeding increased in both study groups from baseline to endline (intervention 27.0 pp, *P*<0.001; comparison 16.1 pp, *P* = 0.039) and their knowledge of exclusive breastfeeding was high at baseline and changed little from baseline to endline.

**Table 3:** Impact of Alive & Thrive on mothers’ knowledge and awareness of key IYCF practices in Kaduna and Lagos States, Nigeria.

	Baseline				Endline				Intervention T_2_-T_1_ pp difference	Comparison T_2_-T_1_ pp difference	Unadjusted DDE^1^ pp (95% CI)	Adjusted DDE^2^ pp (95% CI)
	Intervention		Comparison		Intervention		Comparison					
	%	N/Total N	%	N/Total N	%	N/Total N	%	N/Total N				
**KADUNA**												
**Knowledge**												
Babies should start breastfeeding within 1 hour of birth	50.4	1463/2903	51.5	760/1475	77.4	1800/2327	67.7	1069/1580	27.0***	16.1*	10.8 (-7.9, 29.5)	10.8 (-7.9, 29.5)
Exclusive breastfeeding means giving the baby only breast milk, no other liquids or solids, up to 6 months	91.5	1662/1816	89.2	703/788	92.5	1825/1973	94.3	1251/1327	1.0	5.1*	-4.1 (-11.7, 3.6)	-4.1 (-11.8, 3.6)
Children 6–23 months should receive 4 or more food groups daily	40.4	980/2423	22.8	286/1252	24.5	520/2124	28.2	417/1478	-16.0*	5.4	-21.3 (-42.1, -0.5)*	-21.3 (-42.1, -0.4)*
**Awareness**												
Ever heard about early initiation of breastfeeding	20.5	637/3110	18.6	297/1594	45.4	1099/2421	39.6	640/1618	24.9***	20.9***	4.0 (-7.9, 15.9)	4.0 (-7.9, 15.8)
Ever heard about feeding only breast milk up to 6 months	59.7	1627/3110	59.3	945/1594	83.5	2021/2421	77.4	1253/1618	25.6***	18.2***	7.4 (-7.5, 22.4)	7.5 (-7.5, 22.5)
Ever heard about feeding a variety foods	39.4	1224/3110	34.4	549/1594	58.4	1415/2421	50.6	819/1618	19.1**	16.2***	2.9 (-9.5, 15.3)	2.9 (-9.8, 15.6)
**LAGOS**												
**Knowledge**												
Babies should start breastfeeding within 1 hour of birth	59.9	520/868	62.8	399/635	75.0	1197/1597	73.9	1192/1612	15.0***	11.1*	3.9 (-9.8, 17.7)	3.2 (-9.5, 15.8)
Exclusive breastfeeding means giving the baby only breast milk, no other liquids or solids, up to 6 months	91.0	755/830	89.7	555/619	97.5	1463/1500	96.7	1467/1517	6.6**	7.0***	-0.5 (-7.5, 6.5)	-0.4 (-7.6, 6.8)
Children 6–23 months should receive 4 or more food groups daily	32.2	253/786	44.1	252/572	34.0	509/1496	33.8	501/1482	1.8	-10.3**	12.1 (-1.1, 23.1)*	12.3 (1.0, 23.6)*
**Awareness**												
Ever heard about early initiation of breastfeeding	46.4	421/908	42.1	276/655	65.7	1076/1637	63.6	1046/1644	19.4***	21.5***	-2.1 (-14.3, 10.0)	-2.6 (-14.4, 9.2)
Ever heard about feeding only breast milk up to 6 months	93.5	849/908	95.3	624/655	95.5	1563/1637	96.2	1582/1644	2.0†	1.0	1.0 (-3.1, 5.1)	1.0 (-3.2, 5.2)
Ever heard about feeding a variety foods	85.2	774/908	82.4	540/655	84.8	1388/1637	85.5	1405/1644	-0.5	3.0	-3.5 (-15.2, 8.2)	-2.5 (-13.2, 8.2)

DDE, difference-in-differences estimate; pp, percentage point; T_1_, time 1 (baseline); T_2_, time 2 (endline)

^1^Unadjusted DDE accounts for clustering at the level of the LGA.

^2^Adjusted DDE accounts for clustering at the level of the LGA and adjusts for child’s age and sex.

^†^*P*<0.1,

*P<0.05,

**P<0.01,

***P<0.001

In Lagos, a positive differential impact of the intervention was found for mothers’ knowledge of dietary diversity (unadjusted DDE 12.1 pp, *P* = 0.033; adjusted DDE 12.3 pp, *P* = 0.034) because mothers’ knowledge in the intervention group remained static, while it decreased in the comparison group from baseline to endline (intervention 1.8 pp, *P* = 0.684; comparison -10.3 pp, *P* = 0.002). No impacts were detected for other knowledge variables because mothers’ knowledge increased a similar amount from baseline to endline in both study groups for early initiation of breastfeeding (intervention 15.0 pp, *P*<0.001; comparison 11.1 pp, *P* = 0.042) and exclusive breastfeeding (intervention 6.6 pp, *P* = 0.001; comparison 7.0 pp, *P*<0.001).

Based on responses where mothers were asked if they had ever heard of specific key IYCF practices, in Kaduna, no differential impacts of the intervention were detected on mothers’ awareness of optimal IYCF practices (Table 3). This can be attributed to similar increases in mothers’ awareness from baseline to endline in both study groups for early initiation of breastfeeding (intervention 24.9 pp, *P*<0.001; comparison 20.9 pp, *P*<0.001), exclusive breastfeeding (intervention 25.6 pp, *P*<0.001; comparison 18.2 pp, *P*<0.001), and dietary diversity (intervention 19.1 pp, *P* = 0.001; comparison 16.2 pp, *P*<0.001).

In Lagos, no differential impacts of the intervention were found on mothers’ IYCF awareness. This can be explained by similar increases from baseline to endline in mothers’ awareness of early initiation of breastfeeding (intervention 19.4 pp, *P*<0.001; comparison 21.5 pp, *P*<0.001) and by minimal changes in both study groups for mothers’ awareness of exclusive breastfeeding and dietary diversity.

#### Mothers’ exposure to the intervention

In Kaduna, at endline, the majority of mothers reported exposure to interpersonal communication at health facilities and one-third to one-half were exposed to mass media, while few were exposed to community mobilization activities (Table 4). More mothers in the intervention group were exposed to community mobilization (4.7 pp, *P* = 0.092) and mass media (15.2 pp, *P* = 0.080).

**Table 4:** Mothers’ exposure to Alive & Thrive intervention components and association of exposure with IYCF practices, by state.

	ENDLINE EXPOSURE					ASSOCIATION OF EXPOSURE WITH IYCF PRACTICES^1^		
	Intervention		Comparison		Difference	Early initiation of breastfeeding^2^	Exclusive breastfeeding	Minimum dietary diversity
	%	N	%	N	pp	OR (95%CI)	OR (95%CI)	OR (95%CI)
**KADUNA**								
Interpersonal communication at health facility	80.5	1951	70.6	1143	9.9	2.8 (2.3, 3.4)	3.0 (2.5, 3.5)	2.1 (1.7, 2.6)
Community mobilization	11.0	266	6.3	102	4.7†	1.7 (1.2, 2.4)	1.6 (1.1, 2.4)	1.7 (1.3, 2.2)
Mass media	48.4	1172	33.3	538	15.2†	2.0 (1.7, 2.2)	1.8 (1.5, 2.3)	1.7 (1.4, 2.1)
**LAGOS**								
Interpersonal communication at health facility	94.2	1542	93.6	1538	0.6	1.4 (1.1, 1.8)	1.7 (1.2, 2.4)	1.4 (1.0, 1.8)
Community mobilization	6.4	105	5.3	87	1.1	1.0 (0.6, 1.6)	0.7 (0.4, 1.3)	1.4 (0.9, 2.3)
Mass media	72.9	1193	66.0	1085	6.9†	1.1 (1.0, 1.3)	1.0 (0.7, 1.2)	1.0 (0.8, 1.4)

^1^Associations were tested for intervention and comparison groups combined and account for clustering at the level of the LGA.

^2^Early initiation analyses were adjusted for caesarean section.

^†^*P*<0.1

In Lagos, at endline, nearly all mothers in both study groups were exposed to interpersonal communication and approximately two-thirds to three-quarters were exposed to mass media, while few were exposed to community mobilization. Differences in exposures between the intervention group and comparison group were negligible for interpersonal communication and community mobilization and slightly higher in the intervention group then the comparison group for mass media (6.9 pp, *P* = 0.060).

#### Association of intervention exposures with IYCF practices

In the study groups combined, mothers had increased odds of early initiation of breastfeeding if they received interpersonal communication at a health facility [Kaduna OR 2.8, 95% CI (2.3, 3.4); Lagos OR 1.1, 95% CI (1.1, 1.8)], participated in community mobilization [Kaduna OR 1.7, 95% CI (1.2, 2.4)], or were exposed to Start Strong mass media [Kaduna OR 2.0, 95% CI (1.7, 2.2); Lagos OR 1.1, 95% CI (1.0, 1.3)] (Table 4). Mothers had increased odds of exclusive breastfeeding if they were exposed to interpersonal communication at a health facility [Kaduna OR 3.0, 95% CI (2.5, 3.5); Lagos OR 1.7, 95% CI (1.2, 2.4)], participated in community mobilization [Kaduna OR 1.6, 95% CI (1.1, 2.4)], or were exposed to Start Strong mass media [Kaduna OR 1.8, 95% CI (1.5, 2.3)]. Similarly, mothers had increased odds of minimum dietary diversity if they were exposed to interpersonal communication at a health facility [Kaduna OR 2.1, 95% CI (1.7, 2.6); Lagos OR 1.4, 95% CI (1.0, 1.8)], participated in community mobilization [Kaduna OR 1.7, 95% CI (1.3, 2.2)], or were exposed to Start Strong mass media [Kaduna OR 1.7, 95% CI (1.4, 2.1)].

### COVID endline sensitivity analysis

A sensitivity analysis was conducted to assess whether the COVID-19 pandemic affected endline prevalence estimates for the three main outcomes (Table 5). In Kaduna, minimum dietary diversity decreased before compared with during the pandemic (unadjusted -13.4 pp, *P*<0.001; adjusted -10.0 pp, *P* = 0.003). In Lagos, exclusive breastfeeding increased during the pandemic compared with before the pandemic (unadjusted 11.0 pp, *P*<0.001; adjusted 10.6 pp, *P*<0.001).

**Table 5:** COVID endline sensitivity analysis for IYCF practices, by state.

	ENDLINE				Unadjusted Difference^1^ pp (95% CI)	Adjusted Difference^2^ pp (95% CI)
	Before COVID		During COVID			
	%	N/Total N	%	N/Total N		
**KADUNA**						
Early initiation of breastfeeding	45.6	1445/3116	41.0	342/834	-4.6 (-17.0, 7.8)	-7.4 (-19.9, 5.0)
Exclusive breastfeeding	39.5	414/1047	37.5	144/384	-2.0 (-10.7, 6.7)	-0.9 (-10.4, 8.6)
Minimum dietary diversity	42.2	908/2153	28.8	131/455	-13.4 (-20.0, -6.8)***	-10.0 (-16.5, -3.6)**
**LAGOS**						
Early initiation of breastfeeding	45.6	596/1495	41.0	725/1729	-4.6 (-17.0, 7.8)	-7.4 (-19.9, 5.0)
Exclusive breastfeeding	57.0	227/398	68.0	600/882	11.0 (5.6, 16.4)***	10.6 (5.2, 16.1)***
Minimum dietary diversity	60.6	690/1138	57.0	492/863	-3.6 (-7.7, 0.5)	-1.9 (-6.7, 3.0)

pp, percentage point

^1^Unadjusted difference accounts for clustering at the level of the LGA.

^2^Adjusted difference accounts for clustering at the level of the LGA and adjusts for number of adults in the household, child age (0–5, 6–11, 12–17, 16–23 months), type of cooking fuel, and household food insecurity access category (secure, mild, moderate, severe).

*P<0.05,

**P<0.01,

***P<0.001

A sensitivity analysis of exposures to intervention components before and during the pandemic was also conducted. In Kaduna, a higher percentage of mothers reported exposure to interpersonal communication at a health facility (80.2% vs. 64.6%, difference 15.6 pp, *P* = 0.013), community mobilization (10.3% vs. 3.9%, 6.4 pp, *P* = 0.001), mass media (46.4% vs. 29.6%, 16.9 pp, *P*<0.001) before compared with during the pandemic. In Lagos, a higher percentage of mothers were exposed to community mobilization activities before than during the pandemic (10.0% vs. 2.1%, 7.9 pp, *P*<0.001), whereas interpersonal communication and mass media exposure did not differ before compared with during the pandemic.

## Discussion

This evaluation was designed to measure the impact of IYCF interpersonal communication and community mobilization layered on to IYCF mass media in two states in Nigeria. The assumption was that Alive & Thrive mass media—particularly television and radio ads, billboards, and vehicle ads—would be accessible to women throughout both states, but interpersonal communication and community mobilization would be confined to the intervention group. However, exposure to interpersonal communication did not differ by study group in Lagos or Kaduna. Community mobilization exposure was low in both states, did not differ by study group in Lagos, and was only slightly higher in the intervention group in Kaduna. As a result, we found weak impacts in the difference-in-differences analysis, with an effect of the intervention only on exclusive breastfeeding in Kaduna. DDEs were generally small because most IYCF indicators increased from baseline to endline in both the study groups in both states. Increases in IYCF indicators in the comparison group were likely related to intervention spillover, which is discussed in more detail below. Our exposure analysis supports the link between intervention components and increases in IYCF practices from baseline to endline. Given that Alive & Thrive was the major IYCF activity in collaboration with both state Ministries of Health during this period, it is unlikely that the changes in IYCF practices were brought about by other programs. It is possible that the changes detected in this study are part of a secular trend, which cannot be ruled out in before-after comparisons [33].

To put the findings into context, we compared our results to trends in the Nigeria DHS conducted in 2008, 2013, and 2018, where available [5, 6, 28]. In Kaduna, the prevalence of early initiation of breastfeeding was 38% in 2008 and remained stable at 36% from 2013 to 2018 based on the DHS, whereas it increased in this evaluation in the intervention group from 35% in 2017 to 47% in 2020 and in the comparison group from 35% in 2017 to 41% in 2020. These increases are unlikely to be a secular trend because there was no increase in the previous 10 years. In Lagos, the DHS reported a decline in early initiation from 30% in 2008 to 20% in 2013 and then an increase to 59% in 2018; in this evaluation, it increased in the intervention group from 40% in 2017 to 43% in 2020 and decreased from 44% to 39% in the comparison group. The DHS used a different definition for minimum dietary diversity in 2008, so only 2013 and 2018 are described. In Kaduna, the DHS reported very little change in minimum dietary diversity from 17% to 18% from 2013 to 2017, whereas this evaluation found a large increase in the intervention group from 21% in 2017 to 40% in 2020 and in the comparison group from 19% in 2017 to 39% in 2020. Again, this is unlikely to be a secular trend given the previous lack of change in the DHS. In Lagos, the DHS reported a large increase in minimum dietary diversity from 11% to 41% from 2013 to 2018. This evaluation also found an increase in the intervention group from 40% in 2017 to 59% in 2020 and in the comparison group from 35% in 2017 to 60% in 2020. It is difficult to tell if this is a continuing trend considering the large increase in the DHS. The DHS does not estimate exclusive breastfeeding prevalence at the state level. Exclusive breastfeeding increased nationally from 13% in 2008 to 17% in 2013 and to 29% in 2018. In this evaluation, exclusive breastfeeding increased from 27% in 2017 to 44% in 2020 in the intervention group and from 28% in 2017 to 33% in 2020 in the comparison group in Kaduna. Similarly, exclusive breastfeeding increased from 55% in 2017 to 64% in 2020 in the intervention group and from 59% to 66% in the comparison group in Lagos. Without state-level estimates, it is difficult to know whether the national trend in exclusive breastfeeding detected in the DHS was also occurring in the states. However, the increases in exclusive breastfeeding in both study groups in Kaduna and Lagos suggest that it may be related to the intervention. It is also worth noting that the 2018 DHS took place during the Alive & Thrive implementation period and partly could be picking up changes in the indicators related to the Alive & Thrive intervention in both states.

To our knowledge, the Alive & Thrive impact evaluations in other countries are the only other evaluations of at-scale multicomponent IYCF social and behavior change communication programs in LMICs. The evaluations in Bangladesh, Vietnam, and Ethiopia were designed to compare low-intensity and high-intensity versions of the same intervention [15, 19, 20, 22], whereas the evaluation in Burkina Faso was similar to our evaluation in Nigeria because it compared intervention and control groups [23]. The difference-in-differences estimates from the present study were generally smaller than shown in the Alive & Thrive evaluations in Bangladesh, Vietnam, and Burkina Faso. One major difference between Alive & Thrive in Nigeria and the other countries was the frequency of contacts. Alive & Thrive in Nigeria did not set a target number of contacts or specific timing for contacts, in contrast to Alive & Thrive in Bangladesh and Vietnam. In Nigeria, we did not find an increase in the mean number of times mothers received information on IYCF at health facilities or in the community from baseline to endline. Despite this, we found large increases in IYCF indicators over time. These increases could be explained by women receiving better quality interpersonal communication through Alive & Thrive or messages from multiple sources.

The urban setting of Lagos is another important difference between this study and evaluations of Alive & Thrive interventions in other countries. Our baseline to endline findings suggest that increases in exclusive breastfeeding and minimum dietary diversity may be achieved in a highly urban location, and our exposure analysis indicates that interpersonal communication may have contributed to this. To our knowledge, there are no published evaluations of urban IYCF interventions at scale. We identified two small-scale intervention studies that measured the impact of interventions on exclusive breastfeeding in urban Kenya, but they were not conclusive. One of the studies used a home-based nutrition counseling and support intervention and found no impact on exclusive breastfeeding [34]. The other study showed that a home-based intensive counseling intervention increased exclusive breastfeeding, whereas a low-intensity facility-based intervention was not effective [35].

Mothers’ knowledge and awareness of IYCF is often associated with carrying out the practices, although there are multiple other factors that influence IYCF practices [15–17, 21, 36]. In this evaluation, mothers’ IYCF knowledge and/or awareness generally increased from baseline to endline in Kaduna in tandem with increases in IYCF practices. The picture was more mixed in Lagos. Increases in knowledge of exclusive breastfeeding aligned with increases in the practice from baseline to endline, while increases in knowledge and awareness of early initiation of breastfeeding did not translate into increases in the practice in Lagos. Early initiation of breastfeeding is influenced by many factors other than knowledge, including the woman having a caesarean section and the advice and actions of midwives and nurses present at delivery [37, 38]. In our analysis of the relationship between Alive & Thrive exposures, we found that caesarean section was associated with lower odds of early initiation of breastfeeding and a great percentage of mothers in Lagos had a caesarean section (12% to 15%). In addition, glucose water and infant formula were among the most common prelacteal feeds in Lagos. These are often recommended by midwives and nurses. More research is needed to develop strategies to change norms related to early infant feeding among mothers and health workers in Lagos.

Our exposure analysis showed that most women were exposed to interpersonal communication, followed by mass media, and then community mobilization, which few mothers participated in. The substantial number of mothers in the comparison group who were exposed to intervention components, especially interpersonal communication, suggests that there was some spillover of the intervention to the comparison group. Alive & Thrive staff noted that the Kaduna State Ministry of Health was incorporating IYCF messages into its statewide community-based management of acute malnutrition program. In Lagos, most health services are private, and women often seek care outside of the LGA where they live; women in comparison LGAs may have received IYCF interpersonal communication through their health facility located in an intervention LGA. The Alive & Thrive team noticed that providers trained by the project were often reassigned or moved between health facilities, including between facilities in intervention and comparison LGAs. The team also suspects the state Ministries of Health may have used their resources to implement similar activities and health worker training in the comparison LGAs to keep the states overall from appearing to lag behind on IYCF. In evaluations of large-scale public health programs, intervention spillover to the comparison group is always a risk, especially in a context where the intervention is implemented in collaboration with the local government and the intervention and comparison groups are within the same administrative unit (e.g., state). The fact that state governments may have felt compelled to implement IYCF activities in the comparison LGAs is a testament to the ability of Alive & Thrive and others to generate interest in this important health topic. However, it creates a challenge to determine definitively if the Alive & Thrive intervention had an impact on IYCF knowledge, awareness, and practices.

In addition to spillover of the intervention to the comparison group, this study had several other limitations. The evaluation team was asked to combine two study groups after the LGAs were already randomized, which contributed to imbalances in the sample sizes in the intervention and comparison groups. The evaluation team also was requested to shift from overall estimates, as planned at baseline, to state-level estimates at endline. This change may have resulted in insufficient sample sizes at baseline in some subgroups to adequately estimate state-level impact, particularly for exclusive breastfeeding in Lagos. We chose a geosampling method because of challenges with creating an accurate sampling frame in Nigeria. Although this method was used in other studies in Nigeria and other LMICs [39], we found that geosampling was not ideally suited for enrolling households with narrow eligibility criteria, especially households with a child aged 0 to 5 months. However, we were able to supplement the geosample with random route walks. The COVID-19 pandemic began during our endline data collection and caused us to pause data collection for several months. In our sensitivity analysis, the outcome variables that differed in endline data collected before and during the pandemic were minimum dietary diversity in Kaduna and exclusive breastfeeding in Lagos. It is not possible to determine if these differences are related to the pandemic or to our use of random route walks during the pandemic to fill in gaps in our 0- to 5-month age-group sample. However, given increases in food insecurity globally during the pandemic, it is conceivable that the pandemic contributed to lower dietary diversity among young children in Kaduna. Similarly, during the early months of the pandemic when lockdowns were in effect, more women in Lagos may have been at home with their babies, which could have given them the opportunity to exclusively breastfed.

## Conclusions

This evaluation found limited impacts of the Alive & Thrive intervention on IYCF practices in the difference-in-differences analysis, likely because of intervention spillover to the comparison group. However, the increases in IYCF practices from baseline to endline were substantial and are likely attributable to Alive & Thrive, which was the major implementation partner collaborating with the government and supporting IYCF promotion at scale in both states during the intervention period. The modest levels of early initiation of breastfeeding in both states and of exclusive breastfeeding and minimum dietary diversity, particularly in Kaduna, indicate a need for programs to further improve interpersonal communication in health facilities and during home visits and to include a sustained mass media campaign. A higher prevalence of optimal IYCF practices than measured at endline in this study is necessary to achieve major reductions in child mortality and improvements in child growth and development [1, 39]. As the government of Nigeria continues to implement the IYCF intervention in Kaduna and Lagos and replicate the effort in other states, it should focus on training health workers to carry out facility-based interpersonal communication, as this had the strongest relationship with IYCF practices in both states, and on increasing community outreach and mobilization.

## Supporting information

S1 File Alive & Thrive Nigeria theory of change. (PDF)

S2 File Alive & Thrive Nigeria impact evaluation survey sample sizes for baseline and endline. (DOCX)

S3 File Alive & Thrive Nigeria impact evaluation initial study protocol. (DOC)

S4 File Inclusivity statement. (DOCX)
