# Supplementary material for: Impacts of a social and behavior change communication program implemented at scale on infant and young feeding practices in Nigeria: Results of a cluster-randomized evaluation
Source: PLoS One. 2022 Dec 8;17(12):e0277137. doi: 10.1371/journal.pone.0277137 (PMC9731440; doi:10.1371/journal.pone.0277137)
Supplement: S1 File — (PDF) [file pone.0277137.s001.pdf]

S1. Alive & Thrive Nigeria theory of change

IF WE ADDRESS THESE BARRIERS

Few resources and insufficient priority given to IYCF policies and interventions among policymakers and government workers at all levels

Inadequate health services (resulting in low demand/use) with ineffective integration of IYCF interpersonal communications (IPC) and support during the first two years

Limited knowledge, skills, and motivation of frontline workers to effectively provide IPC and support for IYCF

Lack of social support for recommended IYCF practices among family members, community leaders, and others

Cultural misperceptions and traditional practices resulting in poor IYCF

Insufficient data and knowledge to guide policies and interventions

WITH THESE STRATEGIES

Targeted advocacy and technical policy support at national and state levels to build strategic partnerships for operationalizing new National Social and Behavior Change Communication strategy for IYCF

Improved coverage and quality of IPC for IYCF at facility and community level

Use of data and IYCF capacity building for wide range of health care providers, public, private and informal sectors

Community mobilization reaching ward development committees, religious, community leaders, and others

Mass communication to reach multiple audiences, and address relevant beliefs and misperceptions

Learning, innovation, documentation, and dissemination to stimulate enhanced service delivery, replication and expansion of interventions and policies.

WE WILL ACHIEVE

Additional IYCF resources invested nationally and at the state level

New and improved policies and guidelines supportive of IYCF are in place and supported at national and state levels

Health worker capacity to deliver and coverage of quality IPC increased and demand for services generated

Mothers supported during pregnancy, at delivery, and up to 2 years to feed as recommended

Widespread belief among priority target groups (mothers, grandmothers, health workers, fathers) in recommended IYCF practices

Lessons learned and applied based on assessments and testing innovations

IMPACT

Improved IYCF practices: early initiation of breastfeeding, exclusive breastfeeding, and minimum dietary diversity indicators increased

Newborn and infant lives saved

Improved child heath, growth, and development
