# Supplementary material for: Impacts of a social and behavior change communication program implemented at scale on infant and young feeding practices in Nigeria: Results of a cluster-randomized evaluation
Source: PLoS One. 2022 Dec 8;17(12):e0277137. doi: 10.1371/journal.pone.0277137 (PMC9731440; doi:10.1371/journal.pone.0277137)
Supplement: S2 File — (DOCX) [file pone.0277137.s002.docx]

S2. Alive & Thrive Nigeria impact evaluation household survey sample sizes for baseline and endline

S1a. Minimum number of completed interviews required to measure overall differences between study areas: Baseline^1^

| State | Urbanization | Intervention | | Comparison | | Total |
| --- | --- | --- | --- | --- | --- | --- |
|  |  | 0–5 Months | 6–23 Months | 0–5 Months | 6–23 Months |  |
| Kaduna | Rural | 268 | 268 | 268 | 268 | 1,072 |
|  | Urban | 268 | 268 | 268 | 268 | 1,072 |
| Lagos | Urban | 268 | 268 | 268 | 268 | 1,072 |
| Total | | 804 | 804 | 804 | 804 | 3,216 |

^1^The assumption at baseline was that both states would be combined to generate overall estimates of impact.

S1b. Minimum number of completed interviews required to measure overall differences between study areas: Endline ^1^

| State | Urbanization | Intervention | | Comparison | | Total |
| --- | --- | --- | --- | --- | --- | --- |
|  |  | 0–5 Months | 6–23 Months | 0–5 Months | 6–23 Months |  |
| Kaduna | Rural | 258 | 235 | 245 | 250 | 988 |
|  | Urban | 258 | 235 | 245 | 250 | 988 |
| Lagos | Urban | 618 | 618 | 568 | 568 | 2,372 |
| Total | | 1,134 | 1,088 | 1,058 | 1,068 | 4,348 |

^1^The assumption at endline was that estimates would be calculated for each state.
