# Supplementary material for: Impacts of a social and behavior change communication program implemented at scale on infant and young feeding practices in Nigeria: Results of a cluster-randomized evaluation
Source: PLoS One. 2022 Dec 8;17(12):e0277137. doi: 10.1371/journal.pone.0277137 (PMC9731440; doi:10.1371/journal.pone.0277137)
Supplement: S3 File — (DOC) [file pone.0277137.s003.doc]

**S3. Alive & Thrive Nigeria impact evaluation initial study protocol**

**RESEARCH TRIANGLE INSTITUTE**

**COMMITTEE FOR THE PROTECTION OF HUMAN SUBJECTS**

**Request for Approval of Research Protocol**

**RTI Project/Proposal No.____** XXXXXXXXX**___________________Date__07/16/2016____**

__________________________________________________________________________________

**Title:** Alive & Thrive Nigeria Impact Evaluation

**Sponsor:**

| Check Here If Grant | X |
| --- | --- |

**Project Duration:**

From___04/15/2016__________ To:_____08/30/2019_______________

Month/Day/Year Month/Day/Year

**Date Approval Requested:**______Initial request - 7/20/2016, Second request – 11/21/2016_

Month/Day/Year

**Date Participation of Human Subjects Scheduled to Begin:**____01/09/2017________

Month/Day/Year

**Reason for Review:** (Check One)

|  | Proposal |  |  | Pretest or  Pilot |
| --- | --- | --- | --- | --- |
|  |  |  |  |  |
|  | Renewal |  | X | Full Study  Implementation |
|  |  |  |  |  |
|  | Pre-Award |  |  | Other  (specify) |

Project Leader_____ Valerie Flax ___________________________ Date____11/21/2016______________________________________________________________

**I. STUDY DESCRIPTION**

**A. Type of Study:**

(Check all that apply)

| X |  | Survey |
| --- | --- | --- |
|  |  | Record abstraction |
|  |  | Participation observation |
|  |  | Laboratory experiment or measurement |
|  |  | Device, drug, or procedural trial |
|  |  | Biological specimen collection |
|  |  | Environmental measurement or testing |
| X |  | Other (specify): Focus Groups and In-depth Interviews (IDIs) |

**B. Study Aims:** (200-300 words)

Alive & Thrive (A&T) is an initiative to save lives, prevent illness, and ensure healthy growth and development through improved breastfeeding and complementary feeding practices. As part of A&T’s efforts in Nigeria, an external evaluation will be conducted to rigorously measure the impact of two different A&T intervention models as adapted in Kaduna and Lagos states. These interventions will use different types of communication strategies, including interpersonal communication, community mobilization, and mass media communication. The two intervention models will include community mobilization and will differ in the interpersonal communication strategy. The two strategies are: (1) an intensive interpersonal communication intervention with a full array of messages on IYCF and multiple interactions with women in the target group and (2) a streamlined interpersonal communication approach with a more targeted set of messages and fewer interactions. These two interventions will be compared to mass media communication on infant and young child feeding, which will be provided throughout the two states. The interventions will be implemented by FHI 360 and the impact evaluation will be conducted by RTI International, TNS RMS Nigeria Ltd, and the University of North Carolina. We are asking RTI’s IRB to review the impact evaluation, which RTI is leading, and not the intervention, which is being implemented by FHI 360.

***Table 1*** describes the entities involved in this project and which IRB will cover them. We would like to request that Dr. Kavita Singh at the University of North Carolina would be covered by RTI’s IRB. She will be assisting us with the quantitative data analysis, but will not be involved in data collection and will not have direct contact with study participants. TNS RMS Nigeria Ltd. is our data collection partner in Nigeria. FHI 360 will oversee the implementation of the intervention and FHI’s IRB is reviewing the intervention. They are not asking RTI’s IRB to cover FHI staff. As agreed with FHI’s IRB, RTI’s IRB will review the evaluation.

**Table 1.** IRB Coverage

| Institution | IRB | Status of IRB approvals |
| --- | --- | --- |
| RTI Staff | RTI | Initial review completed 8/22/2016 |
| TNS RMS Nigeria Ltd. Staff | State IRBs in Lagos and Kaduna | Lagos – obtained 8/9/2016  Kaduna – obtained 8/29/2016 |
| UNC-Chapel Hill (Dr. Kavita Singh) | RTI | Initial review completed 8/22/2016 |
| FHI Staff – for the intervention only | FHI | Obtained 11/16/2016 |

The following are the primary and secondary aims of the A&T evaluation in Lagos and Kaduna states:

***Primary evaluation aim:***

- To measure the impact of the two A&T intervention models on IYCF practices among mothers with children 0 to 23 months. We will specifically evaluate:
  - Difference in proportion from baseline to end-line of children 0-23 months who were initiated on breastfeeding within ½ hour of delivery in the intervention arms versus the comparison arm
  - Difference in proportion from baseline to end-line of children 0-5 months who were exclusively breastfed on the previous day in the intervention arms versus the comparison arm
  - Difference in proportion from baseline to end-line of children 6-23 months who were fed the minimum dietary diversity on the previous day in the intervention arms versus the comparison arm
  - Difference in proportion from baseline to end-line of children 6-23 months who were fed the minimum meal frequency on the previous day in the intervention arms versus the comparison arm

***Secondary evaluation aims are to document:***

- The type, quantity, and timing of the implementation of A&T program activities by intervention model
- Coverage levels A&T achieves with different program activities by intervention model
- Extent to which A&T increases knowledge and awareness of optimal IYCF practices among women with children 0 to 23 months and health providers by intervention model
- Extent to which A&T improves the capacity of health providers to counsel mothers on IYCF by intervention model
- Extent to which A&T improves the capacity of stakeholders to implement community-based activities that support optimal IYCF practices by intervention model

**II. STUDY DESCRIPTION**

We will use a cluster-randomized design with three study arms. Local Government Areas (LGAs) in Lagos and Kaduna states (n=39) will receive the mass communication messages and the other two interventions. The interventions will be assigned in randomly selected LGAs. Thirteen LGAs will be included in each study arm stratified by state. Program impact will be measured through baseline and endline surveys conducted in households that have women with at least one child 0 to 23 months and through relevant health service providers. The baseline survey is planned for November 2016 and the endline will be conducted at the same time of year in 2018. Tablets will be used to conduct the household and health service provider surveys. We have selected a probability-based design because it is the most rigorous and allows the causal attribution of differences in observed impacts to the A&T program variants. This is a mixed methods evaluation. The quantitative data will be complemented by qualitative data obtained from different groups targeted by or involved in the program. This application covers baseline, mid-line (qualitative only), and end-line data collection. Procedures for survey and qualitative data collection, data management, and ethics will be the same for all points in time to eliminate any additional sources of variance or confounding factors. Data analysis at baseline will be mainly descriptive with some statistical tests to assess if there are any significant differences between the different study arms, whereas advanced regression models using a difference in differences approach and multiple comparisons will be used to measure impact at end-line.

**A. Sample Size(s):** Sample sizes are described in detail in Table 1.

**Table 1.** Data Sources, Timing of Collection, and Sample Sizes^a^

|  | Baseline | Midline | End-line |
| --- | --- | --- | --- |
| Impact Surveys |  |  |  |
| Completed household screeners with adult household member and household surveys with mothers with a child 0 to 23 months | 5,010 (2,906 Lagos/  2,104 Kaduna) | — | 5,010 (2,906 Lagos/ 2,104 Kaduna) |
| Health Provider Surveys^b^ |  |  |  |
| Completed surveys with health providers | 365 | — | 365 |
| Qualitative Data^c^ |  |  |  |
| In-depth interviews with stakeholders | 32 | 32 | 32 |
| FGDs: mothers with a child 0 to 23 months | 16 groups | 16 groups | 16 groups |
| FGDs: fathers with a child 0 to 23 months | 16 groups | — | 16 groups |
| FGDs: older women with a grandchild 0 to 23 months | 16 groups | — | 16 groups |
| Other |  | | |
| A&T implementation reports, supervision data, and other program implementation documentation | Throughout the intervention period | | |

^a^ This table shows *completed* household and provider surveys; additional households will need to be screened to achieve these sample sizes. Assumptions for sample size calculations are outlined in the following section.

^b^Health provider surveys will be proportionally divided between Lagos and Kaduna.

^c^Qualitative data collection will be evenly divided between Lagos and Kaduna (e.g., 8 FGDs with mothers in Lagos and 8 in Kaduna).

**B. Special Populations** (Check all that apply)

| X |  | None |
| --- | --- | --- |
|  |  | Minors |
|  |  | Newborns |
|  |  | Pregnant |
|  |  | HIV infected |
|  |  | Prisoners |
|  |  | Alcohol, drug, or mental health program clients |
|  |  | Incompetent |
|  |  | Employees (specify)_______________________________________ |
|  |  | RTI Employees, their family member or friends (specify)_________________________________________________ |
|  |  | Other (specify)____________________________________________ |

**C. Sample Selection Procedure(s):** (100-200 words)

***Table 2*** outlines eligibility criteria for all forms of evaluation data collection.

***Randomization and household survey sample selection***

For the cluster-randomized design, LGAs in Kaduna and Lagos states will serve as the clusters. Within each state, LGAs will be matched based on a preset list of criteria (e.g., size of population, urban vs. rural in Kaduna) and then randomly assigned with equal probability to one of the three study arms.

The sampling methodology is based on a geographic sampling design consisting of a multistage stratified clustered random sample. Stratification will be by state, by age group (mothers with 0 to 5 months, 6 to 23 months old children), by urbanicity, and type of intervention at LGAs.

A total of sixteen LGAs in Lagos and 23 LGAs in Kaduna will be covered by the sample. Within each LGA, a GIS grid will be overlaid on the map to divide the LGA into 1 km^2^ grid cells, which will represent the first level of clustering—Primary Sampling Units (PSUs). The maps of the PSUs will be obtained from the National Population Commission, and LandScan 2011 population estimates will be attached to it to provide an estimate of the population size within each unit. PSUs will then be selected using probability proportional to size. Sampled PSUs will be verified as being residential through visual inspection of aerial maps. On average, a total of 20 households will be completed per PSU. To account for the number of households with an eligible women, non-response rates, and the number of PSUs that may be non-residential areas, we estimate a total of 200 PSUs will be required in Lagos (with a reserve sample of 100 spare PSUs), and 140 in Kaduna (with a reserve sample of 100 spare PSUs). A list of spare PSUs will be provided to the field staff in case the main PSUs are not accessible (e.g., gated communities) or if the targeted sample size is not achieved.

PSUs will be further subdivided into 50 m^2^ cells in urban areas, 100 m^2^ cells in peri-urban areas, and 150 m^2^ in rural areas, creating the secondary sampling units (SSUs) at the second stage of sample selection. The SSUs are classified as either having residential dwellings or containing no residences (e.g., parks, parking lots, highways). Then a systematic sample of two main SSUs and two spare SSUs will be selected from the residential SSUs. All households within the selected SSUs will be included in the sample for screening, except in cases where the SSU contain­­s buildings with more than three floors of living quarters. In this case, a systematic sample of floors will be selected, and a census of households within these floors will be conducted.

To maintain comparability, the same PSUs selected at baseline will be used at endline. Although we will be visiting the same sampling units, we are unlikely to have the same households or respondents within households because of the eligibility to participate in the survey and population mobility especially in urban areas. Geo-location of selected clusters and households will be captured in the tablets and used along with demographic information to conduct the spatial analysis.

***Health provider sample selection***

For health provider surveys, we plan to interview three types of providers: (1) facility-based providers involved in IYCF promotion, (2) patent medicine vendors/community pharmacists, and (3) traditional birth attendants. These types of providers have been selected because A&T expects to provide them with training on IYCF as part of the intervention. The total provider sample in each state will be approximately equally allocated across facility-based providers and non-facility-based providers (i.e., patent medicine vendors, pharmacists, and traditional birth attendants).

For the facility-based providers, we will collaborate with state- and LGA-level Ministry of Health representatives to generate lists of public and private health facilities in intervention LGAs. We will stratify these lists by public/private and size of facility and then select a random sample of facilities using equal allocation. At each selected facility, we will ask the in-charge to name the providers who are involved in IYCF counselling or promotion. At each selected facility, we will ask the in-charge to name the providers who are involved in IYCF counselling or promotion. We will then randomly select one person to complete the survey per selected facility.

For the non-facility-based providers, the sample will be equally allocated across patent medicine vendors, pharmacists, and traditional birth attendants. Comprehensive list frames may not be available for all three types of non-facility-based providers in all LGAs. In LGAs where a list frame is available, a simple random sample of providers will be selected. In LGAs where a list frame is not available, two of the main PSUs from the household study will be randomly selected, one of which will be enumerated during fieldwork and the other will be held in reserve as a backup PSU if an insufficient number of providers was identified in the first PSU. Once the enumeration has been completed, a simple random sample of providers will be selected from the lists using a random number generator. The total number of enumerator providers per LGA will be recorded for calculation of the sampling weights.Each selected health provider will be contacted three times for an interview before being replaced by another randomly selected individual in the same category.

**Table 2.** Eligibility Criteria

| Type of Data Collection | Type of Participant | Eligibility Criteria |
| --- | --- | --- |
| Impact Surveys | | |
| Household surveys | Mothers with young children | Female; 15–49 years; 15-17 year olds must be married; either has a child 0–5 months or 6–23 months; if a woman has a child in both age groups, questions will be asked about the youngest child |
| Provider Surveys | | |
| Health provider surveys | Health provider | Trained health provider in government or private clinic or patent medicine vendor, community pharmacist, or traditional birth attendant; ≥18 years |
| Qualitative Data | | |
| In-depth interviews | Stakeholders | ≥18 years; Ward Development Committee members, religious leaders, NGO workers, or other groups that will be involved in community IYCF mobilization |
| FGDs | Mothers with young children | Female; 15–49 years; 15-17 year olds must be married; has a child 0–23 months; no prior participation in data collection activities for this evaluation; exclude health providers |
| FGDs | Fathers | Male; ≥18 years; has at least one child 0–23 months; no prior participation in data collection activities for this evaluation; exclude health providers |
| FGDs | Older women | Female; has at least one grandchild 0–23 months; no prior participation in data collection activities for this evaluation; exclude health providers |

***Qualitative sample selection***

Working with the A&T team and local authorities, we will develop lists of relevant stakeholders in intervention LGAs who already are or will be involved in social mobilization related to IYCF for recruitment for in-depth interviews. We will group the stakeholders by intervention arm, type of organization (e.g., Ward Development Committee and NGO), and urban/rural location (in Kaduna state only) and will select equal numbers from each intervention and type/location of stakeholder.

For FGDs with mothers, husbands, and grandmothers, we will randomly select 4 LGAs within each intervention arm in each state and 1 ward within those LGAs. In the selected wards, we will use additional criteria to purposively select participants that represent potentially important social differences (e.g., education level, income). For example, in Lagos, we may have some FGDs in urban slum areas and some in areas with higher socioeconomic status. In Kaduna, we will conduct FGDs in both urban and rural areas. The recruitment team will use the screening and recruitment form for FGDs to identify eligible individuals and invite them to participate For the FGDs, we will exclude participants across categories who are related to one another (e.g., we will not select husbands of women who participated in the mothers’ FGDs). We will also exclude health providers.

1. **Participant Recruitment Procedures:** (50-100 words)

***Household surveys***

At each sampled household, interviewers will conduct a household screener to obtain information on the residents of the household and identify mothers (15-49 years) with a child 0 to 23 months who are household residents. An adult present in the household at the time of listing will be the point of contact with the listing team. The team will preferentially do the screener with the head of household or senior female household member. The purpose of the household screener is to determine if the household contains a woman with a child of the appropriate age (either 0 to 5 months or 6 to 23 months). It is possible that in some households the person who completes the household screener and the mother who participates in the survey will be the same person. If more than one eligible household member has a child in the same age range, we will randomly select from among the eligible individuals. The interviewer will conduct the screener, check eligibility, recruit, and conduct informed consent. Interviewers will be instructed to visit eligible households at least three times before excluding it from the sample.

***Health Provider Surveys***

Providers will be recruited in health facilities (facility-based providers), in pharmacies or shops (pharmacists and patent medicine vendors), or at their homes (traditional birth attendants). Each selected facility-based or community-based health provider will be screened by interviewers to check for eligibility. If the individual is eligible, the interviewer will explain the purpose of the study and invite the provider to participate. Interviewers will visit selected providers at least three times before excluding them from the sample.

***Focus Groups and IDI with Stakeholders***

Participants for IDIs with stakeholders will be identified with assistance from Ward or village leaders. Identified stakeholders will be contacted in person or by phone by the recruitment team and checked for eligibility. If they agree to participate, an appointment will be set for the interview. The interviewer will conduct informed consent and the interview will take place in a private location.

A recruitment team will visit the target areas within the selected LGAs for qualitative data collection. In urban areas, they will select buildings or households along pre-identified streets and inquire about the presence of the target audience. In rural areas, they will contact village leaders and ask for their assistance in identifying participants. Potential participants will be visited in their homes by a recruitment team member, who will use the screening form to check for eligibility, recruit and consent the individual, and tell them the time and place for the FGD. FGDs will be held in rented halls or empty community spaces, such as schools that are not in session.

**III. INFORMED CONSENT.**

**A. Type:** Check One

|  |  | Written not signed |
| --- | --- | --- |
|  |  | Written and signed |
|  | X | Verbal not signed |
|  |  | Verbal and signed |
|  |  | Both verbal and written |

**B. Informed Consent Procedures** (200-300 words)

See verbal consent forms for each data collection instrument. We are using an oral consent process because about half of the women in Nigeria are not literate. In this setting, it is culturally appropriate for the interviewers to read the consent form and then ask if the person is willing to participate. For all types of participants included in this study, the consent form will be read aloud, the interviewer will answer any questions the participant has about the study or study procedures, and then ask the participant if he or she is willing to participate in the study. The interviewer will then mark on the consent form whether the person agrees or does not agree to participate. If there is no verbal agreement, the interviewer will discontinue the process and move on to the next eligible individual. If the person consents to participate, the interviewer will sign the paper copies of the form and the participant will be offered a copy of the consent form, which includes the contact information for FHI 360 and the local IRBs. One paper copy of the signed consent form will be kept by TNS as proof that informed consent was obtained.

According to the Nigerian constitution, Section 29 (1) (a) “full age” means the age of eighteen years and above; (b) any woman who is married shall be deemed to be of full age. Under this law, we can enroll married women 15-17 years of age as adults, so parental consent will not be required.

| X | Copy of Consent with Consent Form Checklist Attached |
| --- | --- |

C. Individual Participant Burden

|  | 2 | . | 0 | **Hours - Participant Surveys and Focus Groups** |
| --- | --- | --- | --- | --- |
|  | 1 | . | 0 | **Hours – Healthcare Provider Surveys and IDIs** |

**D. Participant Compensation**

Participants in the household surveys will not receive any incentives. Health providers will receive incentives worth 2000 Nigerian naira and in-depth interview and FGD participants will received incentives worth 1500 Nigerian naira. (1 U.S. dollar = 293 Nigerian naira)

**E. Number of Recontacts:**

| X |  | None – for most participants |
| --- | --- | --- |
| X |  | One – possibly for some participants |
|  |  | Recontacts |

It is unlikely that we will recontact household survey participants. The household surveys are cross-sectional. We will use the same geographical areas at both baseline and endline, but it is unlikely that the same women will be interviewed at both timepoints due to the eligibility criteria (must have a child < 24 months each time and surveys are 2 years apart). Women who participate in household surveys will be excluded from FGDs. We will ensure that we do not include the same FGD participants at more than one time point. It is possible that some health providers for surveys and some stakeholders for in-depth interviews could be contacted at both baseline and endline.

**F. Future Contacts:**

|  | Future contact is planned |
| --- | --- |
| X | No future contact is envisioned |
|  | Future contact might or could be considered |

**IV. DATA COLLECTION PROCEDURES**

**A. Type:** (Check all that apply)

| X | **Survey** |  | **Biological Specimen** |
| --- | --- | --- | --- |
|  | NA |  | NA |
|  | Mail |  | Invasive |
|  | Anonymous |  | Noninvasive |
|  | Personal Interview |  |  |
|  | Self-administered Questionnaire |  | **Device, Drug, or Procedure Trial** |
|  | Telephone Interview |  | NA |
| X | Other (specify) |  | Invasive |
|  | IDIs and focus groups |  | Noninvasive |
|  | **Record Abstraction** |  | **Laboratory Experiment or Measurement** |
|  | NA |  | NA |
|  | File review |  | Psychological |
|  | At agency or facility |  | Physical invasive |
|  | Computer |  | Physical noninvasive |
|  | Request records from agency or  facility |  | Focus group |
|  | Other (specify) |  | Other (specify) |

**B. Description of Procedures:** (200-300 words)

All data collection tools will be translated into Yoruba and Hausa languages. Translation will be done by two translators working independently, and a third translator will check and harmonize areas of disagreement. Translated and English versions of the data collection tools have been submitted to the Lagos and Kaduna State IRBs in Nigeria. Final versions of the translated tools will be submitted to RTI’s IRB prior to beginning data collection. Data collection tools and procedures will be pretested in localities where data will not be collected but that have similar characteristics to the study sites. Training for data collectors and supervisors, including pilot testing of the instruments, will be conducted prior to each phase of data collection. Training for surveys and qualitative data collection will be conducted separately. Each group will receive 5 days of training. Refresher training for qualitative data collection will be shorter (e.g., 3 days) if the same interviewers from baseline are used at midline and endline.

There will be 4 teams of survey data collectors in each state. Each team is comprised of 5 interviewers and 1 supervisor. The LGAs will be grouped by proximity and evenly distributed among the teams. Supervisors will conduct field observations of interviewers to ensure that sampling, recruitment, and interview administration procedures are being followed. Quality control officers will conduct back checks on 15% of completed household and health provider surveys to verify that an interview was conducted and to check the reliability of responses. We will also monitor the quality of the survey data through reviews of weekly datasets and paradata (from a case management system that allows field staff to record information on each contact attempt).

Data for baseline and end-line household and health provider surveys will be collected using mobile electronic devices, specifically Samsung Galaxy tablets that have the nField app. Data are uploaded daily from tablets to a database on a secure server. The database stores all data collected through the questions in the questionnaires, including some variables like date, time, location, and name of the interviewee and interviewer. Only researchers and data managers will have access to the database. The data collection team and IT team will have access to the tablets.

Interviews will be administered by trained data collectors. All routing and skip instructions will be built into the program for the survey prior to data collection, thus reducing data cleaning post-fieldwork. For questions where participants may specify an “other” response, data will be coded following the surveys using code frame developed from 30% of verbatim responses from each state.

In-depth interviews and FGDs will be conducted by moderators/interviewers experienced in qualitative data collection. FGDs will have 6-8 participants and will be led by a moderator, who will be assisted by a note taker. There will be one qualitative team containing two members per state. Qualitative data will be captured using digital voice recorders. Verbatim transcripts in the local language will be prepared from the voice recordings and then translated into English. A random sample of 10% of the English translations will be verified against the local language transcripts, which are considered source documents.

We will try to ensure that interviews take place in a private location, whenever possible. For women being interviewed at home, the interviewer will ask them to find a quiet place in the household where other individuals will not be present. This may be challenging in some households and the interviewer will need to signal to the woman that if others are present her responses will not be fully confidential. Interviewers will be trained in techniques for politely asking other people who enter the space during the interview to leave.

Facility-based health workers will be interviewed in a private office at the health facility. Patent medicine vendors will be interviewed in a store room behind the shop, if available, or in another private location of their choosing. Traditional birth attendants will be interviewed in their homes. Stakeholders will either be interviewed in their homes or offices, if available. For all interviews that take place in individuals’ homes, the interviewers will attempt to keep the interview private as described above for interviews with women. FGDs will be conducted in a private location, such as an empty classroom after school hours or a rented hall.

**V. POTENTIAL RISKS**

**A. Type:** (Check one or more)

|  | None |
| --- | --- |
|  | Minimal physical |
| X | Minimal psychological/social/legal |
|  | Substantial physical |
|  | Substantial psychological/social/legal |

**B. Description of Physical Risks:** (200-300 words)

There are no physical risks to participating in this study.

**C. Description of Psychological/Social/Legal Risks:** (100-200 words)

There are minimal psychological, social, or legal risks to participating in this study. It is possible that participants may feel uncomfortable answering some questions, although the data collection tools do not contain sensitive questions. Participation is voluntary, and respondents can choose not to answer any of the interviewer’s/moderator’s questions. Participants will be assured of confidentiality. We will make all possible efforts to ensure that tablets are secure, consent forms are transferred and stored securely, and data is collected privately.

Breastfeeding is the norm in Nigeria and is considered a normal part of motherhood. There are no religious norms or taboos related to breastfeeding that would put any participants at risk if they shared information about breastfeeding during interviews or FGDs. Given the nature of the study, there is no risk to having religiously diverse participants in FGDs. Further, it is likely that most FGDs will be composed entirely of members of one religion or another because these divisions are related to geographic location and language.

**VI. PROTECTION OF SUBJECTS:**

**A. Guarantees:**

|  | Anonymity (no link between individual and data is possible) |
| --- | --- |
| X | Confidentiality (RTI guarantee only) |
|  | Confidentiality (RTI & other guarantee) (specify) |
|  |  |
|  | ________________________________________________________ |

**B. Types of Procedures Provided to Reduce or Alleviate Risks**

|  | Maintenance or environmental cleanup or correction |
| --- | --- |
|  | Psychological counseling |
|  | Medical treatment |
| X | Other (specify) |
|  | Participation is voluntary, and respondents can choose not to answer any of the interviewer’s/moderator’s questions. |
|  | ________________________________________________________ |

**C. Description of Procedures to Reduce or Alleviate Risks:** (100-200 words)

Participation is voluntary, and respondents can choose not to answer any of the interviewer’s/moderator’s questions.

**D. Description of Security Measures:** (50-100 words)

All of TNS’ survey data collection devices are password protected and all other apps not needed for data collection are blocked. All devices are loaded with a data security app called Air Watch, which makes it possible to track device location, remotely wipe data in case of theft or loss and restrict unauthorized access to ensure data security. The captured data is encrypted to ensure confidentiality while the data are in the tablet and as they are transferred to TNS’ server.

Each survey interview collected on tablets will be uploaded to TNS’ data collection server once completed. If there are connectivity issues in the field, the data will be uploaded at the end of the day. Final data will be downloaded from the data collection server and stored safely as raw data in local TNS servers once the fieldwork is done. The surveys will be deleted from the data collection servers; hence, no one apart from data management personnel can access the data. The final cleaned file that is shared with RTI will only be saved in the secure TNS network. All data stored in the tablets will be deleted. Data from the field will be saved in the TNS backup server. RTI also maintains a secure network, so any data transferred to RTI for data collection monitoring and analysis will be accessible to project staff only.

Qualitative data collected on digital voice recorders will be downloaded to one or more encrypted laptops daily and deleted from the recorders. Voice recordings will be backed up on encrypted flash or external hard drives and transferred to TNS’ server as soon as possible.

Paper copies of signed consent forms will be stored in a locked room at TNS offices and destroyed at the end of the study.

**VII. BENEFITS**

**A. Information Provided to Study Participants**

| X | No direct benefit |
| --- | --- |
|  | Medical or physical data (e.g., serum levels) |
|  | Social data (e.g., eligibility for service) |
|  | Psychological data (e.g., test scores) |
|  | Environmental data (e.g., toxicity levels) |
|  | Other (please specify) |
|  | ______________________________________________________ |

| Brief description:_______________________________________________________________ |
| --- |
| _______________________________________________________________________________ |

**B. Services Provided to Study Participants:**

| X | No direct services provided |
| --- | --- |
|  | Medical or rehabilitation treatment |
|  | Social/economic service |
|  | Psychological counseling |
|  | Environmental cleanup or correction |
|  | Other (please specify) |
|  | ______________________________________________________ |

| Brief description:_______________________________________________________________ |
| --- |
| _______________________________________________________________________________ |

1. **Other Benefits:** (please describe)

There are no other benefits to participation.

# VIII. RISK/BENEFIT RATIO

**A. Type:**

|  | No risk/no individual benefit |
| --- | --- |
| X | Minimal risk/no individual benefit |
|  | Minimal risk/minimal individual benefit |
|  | Minimal risk/substantial individual benefit |
|  | Substantial risk/substantial individual benefit |
|  | Substantial risk/substantial research/society benefit |

**B. Weighing of Risk/Benefit:** (200-300 words)

There is no direct benefit to study participants. There are minimal risks to study participants. No questions are sensitive in nature. Participation is voluntary, and respondents can choose not to answer any of the study questions. The findings from this study will be used to help improve A&T and other programs globally that promote infant and young child feeding practices at scale.

**IX. SPECIAL ISSUES**

**A. Type of Issue or Risk:**

|  | None |
| --- | --- |
| X | Collaborative research |

| X |  | RTI is prime contractor |
| --- | --- | --- |
|  |  | RTI is subcontractor |
|  |  | Other (please specify)_______________________________ |

**B. Discussion of Special Issues and Approach to Minimize Risks:** (200-300 words)

We do not anticipate any other special issues or risks associated with this study.

**X. NEEDS FOR FUTURE REVIEW**

|  |  | Pre-Award | Date | __________________ |
| --- | --- | --- | --- | --- |
|  |  | Pretest/Pilot | Date | __________________ |
|  |  | Full Study Implementation | Date | __________________ |
| X |  | Renewal | Date | Yearly until publications are completed – at least through 2019 |
|  |  | Other (please specify) | Date | __________________ |
